# Intragastric Balloon Therapy: Short-Term Gains and the Quest for Long-Term Success

**DOI:** 10.5152/tjg.2026.25783

**Published:** 2026-02-23

**Authors:** Yavuz Özden

**Affiliations:** Department of Gastroenterology, Kayseri City Hospital, Kayseri, Türkiye

Dear Editor,

I read with considerable interest the prospective single-center study by Yüksel et al, which evaluated 6-month outcomes of endoscopic intragastric balloon (IGB) therapy and demonstrated significant short-term reductions in body weight, body mass index, and abdominal fat compartments assessed using computed tomography.^1^ The low incidence of adverse events further corroborates the procedural safety of IGB when administered to appropriately selected patients.

Beyond confirming short-term efficacy, however, the study raises several clinically and conceptually relevant issues concerning the interpretation of outcomes, external validity, and the evolving role of IGB therapy within contemporary obesity management strategies.

From a methodological perspective, the 6-month follow-up period essentially reflects the active treatment phase during which the balloon remains in situ, rather than a true post-intervention durability phase. While this duration is adequate to demonstrate weight loss driven predominantly by mechanical satiety, it does not permit robust conclusions regarding sustained metabolic benefit or long-term weight maintenance. Prior investigations have consistently reported heterogeneous post-removal weight trajectories, with a substantial propensity for weight regain in the absence of structured post-procedural interventions.^2^^,^^3^ Accordingly, 6-month outcomes should be interpreted primarily as evidence of short-term physiological responses rather than definitive therapeutic success.

In terms of metabolic interpretation, although the reported reductions in visceral and subcutaneous fat thickness are clinically relevant, the absence of concomitant biochemical follow-up, such as glycated hemoglobin, lipid parameters, or validated indices of insulin resistance, constitutes a notable limitation. Accumulating data indicate that reductions in abdominal adiposity do not uniformly translate into proportional improvements in glycemic control or cardiometabolic risk, particularly for interventions whose primary mechanism is gastric volume occupation.^4^ In an era increasingly shaped by metabolically driven treatment objectives, future investigations of IGB therapy would benefit from systematic biochemical monitoring to more accurately delineate true metabolic efficacy.

With respect to external validity, the predominance of the single-balloon system within the study cohort warrants careful consideration. Approximately 90% of the patients were treated with the same device,^1^ a pattern that likely reflects real-world practice in self-pay healthcare settings. Nevertheless, such device homogeneity inherently limits the generalizability of the findings to IGB therapy as a therapeutic class. Device-specific attributes, including adjustability, filling volume, and material composition, may meaningfully influence tolerability, early removal rates, and patient adherence. Consequently, outcomes derived from largely single-device cohorts may reflect system-specific performance rather than universally applicable IGB efficacy, underscoring the need for real-world data to better define the clinical positioning of IGB therapy within contemporary obesity management.^5^

More broadly, these findings prompt a reconsideration of how IGB should be positioned within modern obesity treatment algorithms. The therapeutic landscape has evolved substantially with the widespread implementation of glucagon-like peptide-1 receptor agonists and related agents, which provide clinically meaningful and durable weight loss accompanied by well-established metabolic benefits.^6^ In parallel, endoscopic sleeve gastroplasty has demonstrated superior long-term durability compared with space-occupying devices, albeit at the cost of greater technical complexity.^7^

Within this evolving context, IGB may be better conceptualized not as a stand-alone definitive intervention but as a strategic and time-limited component of a stepwise, multimodal obesity management paradigm. Extending follow-up beyond balloon removal and comparing different balloon systems may help clarify the true durability of IGB therapy. Integrating IGB into multidisciplinary care pathways may further reduce post-intervention weight regain.^8^ Such an approach aligns more closely with contemporary frameworks that recognize obesity as a chronic, relapsing disease requiring longitudinal management.^9^

In conclusion, Yüksel et al provided valuable prospective data confirming the short-term safety and efficacy of IGB therapy. Viewed from this perspective, the study not only reports short-term efficacy, but also provides a timely opportunity to reconsider the role of intragastric balloons in an era increasingly shaped by durable, metabolically driven obesity therapies.^10^ Their findings highlight both the potential and inherent limitations of balloon-based interventions. Rather than viewing IGB as a transient mechanical solution, current evidence supports its repositioning as a bridging modality within a broader multidisciplinary obesity management strategy aimed at achieving durable metabolic and clinical benefits.

Sincerely,

## Data Availability

The data that support the findings of this study are available on request from the corresponding author.
